# Comparative structure and biomechanics of plant primary and secondary cell walls

**DOI:** 10.3389/fpls.2012.00204

**Published:** 2012-08-22

**Authors:** Daniel J. Cosgrove, Michael C. Jarvis

**Affiliations:** ^1^Department of Biology, Pennsylvania State UniversityUniversity Park, PA, USA; ^2^School of Chemistry, University of GlasgowGlasgow, UK

**Keywords:** cellulose, creep, deformation, growth, primary cell walls, secondary cell walls, xyloglucan

## Abstract

Recent insights into the physical biology of plant cell walls are reviewed, summarizing the essential differences between primary and secondary cell walls and identifying crucial gaps in our knowledge of their structure and biomechanics. Unexpected parallels are identified between the mechanism of expansion of primary cell walls during growth and the mechanisms by which hydrated wood deforms under external tension. There is a particular need to revise current “cartoons” of plant cell walls to be more consistent with data from diverse approaches and to go beyond summarizing limited aspects of cell walls, serving instead as guides for future experiments and for the application of new techniques.

## INTRODUCTION

Primary and secondary cell walls are microfibril-based nanocomposites that differ in the arrangement, mobility and structure of matrix polymers, the higher-order organization of microfibrils into bundles and discrete lamellae, their rheological and mechanical properties, and their roles in the life of the plant. Wall structure has sometimes been compared with that of fiberglass – a plastic matrix reinforced by glass-fibers – but this analogy fails to account for the complex rheological behavior of plant walls. In this mini-review we summarize recent insights linking the molecular organization of cell walls with their mechanical and rheological properties.

Primary cell walls are synthesized during growth and typically are relatively thin, pliant, highly hydrated structures. The primary wall must be *strong* to withstand the tensile forces arising from turgor pressure, *extensible* to allow wall stress relaxation which motivates cell water uptake and physical enlargement of the cell (Hamant and Traas, 2010), and *incorporative*, meaning capable of linking newly deposited wall polymers into the load-bearing structure. These properties partly derive from the physical structure of the wall, but they also involve dynamic actions by expansin and xyloglucan endotransglycosylase/hydrolase (Cosgrove, 2005; Eklof and Brumer, 2010).

Secondary cell walls provide strength and rigidity in plant tissues that have ceased growing. Any tall terrestrial plant requires stems with bending strength and with water-conducting tissues that can withstand negative pressures (Koch et al., 2004; Speck and Burgert, 2011). Secondary walls therefore need compressive as well as tensile strength, but not extensibility. Nevertheless, under some conditions they can undergo various deformation processes that resemble, to some degree, primary cell wall growth.

## STRUCTURE AND BIOMECHANICS OF GROWING WALLS

Primary walls are comprised of 15–40% cellulose, 30–50% pectic polysaccharides, and 20–30% xyloglucans and lesser amounts of arabinoxylans and structural proteins, on a dry weight basis, structured into one or more lamellae. The pectic polysaccharides are particularly important for wall hydration, which is essential for the slippage and separation of cellulose microfibrils during expansive growth. Notable deviations from this generic composition include the primary walls of:

- grasses, where arabinoxylans constitute most of the matrix; mixed-linkage glucans transiently comprise 10–20% of wall mass (Carpita et al., 2001; Gibeaut et al., 2005);
- celery and sugar beet parenchyma, which are rich in cellulose and pectin, but have little hemicellulose (Thimm et al., 2002; Zykwinska et al., 2007b).

Understanding how mechanical performance emerges from molecular structure – particularly the mechanism of cell wall expansion – has been a key *raison d’être* of primary cell wall models for decades (Keegstra et al., 1973; Preston, 1974; Cosgrove, 2005), yet despite significant progress we still have a long way to go to achieve the “consistency of molecular structure with the physical properties of the walls during growth” (Carpita and Gibeaut, 1993).

Studies of the viscoelastic properties of primary walls – summarized in numerous reviews (Preston, 1974; Cosgrove, 1993; Geitmann, 2010) – show that unaided viscoelastic extension of walls decays rapidly, amounting to a few % extension, whereas in the living plant it continues for hours or days and results in extensions of 50–100% or more. Normal cell wall expansion is not a simple result of the wall’s constitutive properties but results from continuous action by the cell or by wall-associated proteins. It follows that measures of wall viscoelasticity may correlate with wall structure at any given instant, but these physical measures generally do not encompass this active aspect of wall extensibility, which has sometimes been described as chemorheological creep, meaning polymer creep that depends on chemical or enzymatic modification (Ray and Ruesink, 1962). The gap between *in vitro* and *in vivo* extensibility is at least partly filled by the action of expansins, which induce stress relaxation and sustained creep of cell walls (Sampedro and Cosgrove, 2005). Although we know expansin structure in atomistic detail (Yennawar et al., 2006), the details of its loosening action on the wall remain enigmatic, in part because our understanding of the molecular architecture of the primary wall is incomplete.

### CONVENTIONAL VIEW OF XYLOGLUCAN’S ROLE IN WALL MECHANICS

**FIGURE 1:**
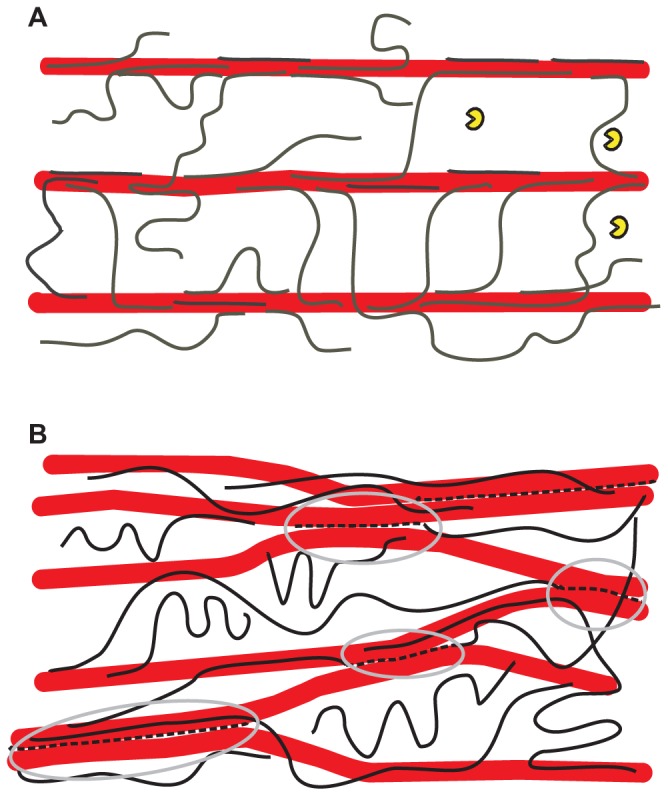
** Alternative hypothetical architectures of cellulose– xyloglucan networks in primary cell walls**. **(A)** The tethered network model in which xyloglucans (black lines) fully coat the surfaces of cellulose microfibrils (larger red rods) and additionally span the 20–40 nm gap between adjacent cellulose microfibrils as load-bearing tethers. The distance between microfibrils is large enough to permit penetration of a xyloglucanase-specific endoglucanase (yellow “Pacman” symbols) of the type used by Park and Cosgrove (2012a). This model is based a variety of results (Scheller and Ulvskov, 2010; Hayashi and Kaida, 2011) but is still hypothetical and recent work is at odds with it. In this model, the primary means of cell wall expansion is by cutting the xyloglucan tethers or by destabilizing the xyloglucan–cellulose interaction, resulting in wall stress relaxation and yielding. The directionality of growth depends on the net orientation of cellulose microfibrils (Baskin, 2005; Kerstens et al., 2001). **(B)** A revised architecture based on the enzyme/biomechanics analysis of Park and Cosgrove (2012a), in which the load-bearing xyloglucan (broken black lines highlighted by gray ellipses) is a minor fraction of the total xyloglucan and is trapped between microfibrils, so it is not accessible to xyloglucan-specific endoglucanase. By this arrangement the xyloglucan glues microfibrils into a network of microfibril bundles which serve to protect it from lytic action by xyloglucan-specific endoglucanases as well as xyloglucan endotransglycosylase/hydrolase. The gray circles demark the limited region that bear the static tensile forces generated by turgor pressure. An arrangement as shown here could account for the lack of wall loosening caused by xyloglucan endotransglycosylase/hydrolases (Saladie et al., 2006; Maris et al., 2009; Miedes et al., 2011).

According to current depictions of primary walls, cellulose microfibrils are coated by xyloglucans and tethered by them to form a load-bearing network, with pectins functioning as a co-extensive, space-filling matrix that separates the microfibrils. This “tethered network model” (**Figure 1A**) might exhibit turgor-driven yield and creep resembling real primary cell walls if xyloglucan binding was dynamic and reversible (Veytsman and Cosgrove, 1998; Dyson and Jensen, 2010). However, if xyloglucan bound cellulose tightly and irreversibly, as appears to be true, this network would behave more like a viscoelastic solid, with retarded elasticity due to the time needed for unfolding of xyloglucan tethers and viscous movement of the cellulose microfibrils within the pectic matrix (Abasolo et al., 2009). On the other hand, there is some doubt that xyloglucan–cellulose binding has sufficient strength to withstand the tensile forces in the wall (Thompson, 2005). This issue seems an open question that might be addressed by molecular dynamics simulations (Bergenstrahle et al., 2009). A thorough analysis is complicated by microfibril structure which has distinct hydrophobic and hydrophilic surfaces as well as disordered regions, which likely differ in xyloglucan binding. Furthermore, xyloglucan may be entrapped within microfibrils or bundles of microfibrils, further complicating such analysis.

The elastic behavior of a multi-lamellate version of the tethered network has been modeled by a finite-element simulation, absent the pectin component (Kha et al., 2010). A key advantage of this approach is that the spatial distribution and range of tensile loads can be calculated for individual tethers. This may be important for extending the model to incorporate viscoelastic extension and creep behavior of walls in a realistic way.

Using another approach, Dyson et al. (2012) modeled the effects of stretch-dependent cross-link breakage on cell wall-yielding behavior, incorporating potential effects of expansin and xyloglucan endotransglycosylase/endohydrolase. The results resemble Lockhart’s semi-empirical equation in which the rate of wall expansion is given by φ (*P* - *Y*), that is, extensibility φ times turgor pressure *P* in excess of the yield threshold *Y* (Lockhart, 1965). Moreover, the model offers a molecular interpretation of φ as due to the pectin viscosity and *Y* as related to the density of xyloglucan tethers. These are testable predictions.

### GROWING DOUBTS ABOUT XYLOGLUCAN’s ROLE

Despite the widespread acceptance of the tethered network model, recent results have led to some doubts about its correctness. As mentioned above, some walls contain little hemicellulose. Attempts to measure xyloglucan bound to cellulose by ^13^C-NMR in intact *Arabidopsis* and mung bean walls concluded that < 10% of the cellulose surface is coated with xyloglucan, contrary to expectations of the dominant model (Bootten et al., 2004; Dick-Perez et al., 2011). Much of the xyloglucan interacted with pectins, which showed extensive interactions with cellulose. Neutral pectins bind to cellulose *in vitro*, although less avidly than does xyloglucan (Zykwinska et al., 2007a, 2008).

Another blow to the tethered network model came from analysis of mutants defective in xyloglucan synthesis which displayed only modest growth defects despite the complete absence of xyloglucan (Cavalier et al., 2008; Zabotina et al., 2012). Further analysis showed that pectins and xylans assumed greater biomechanical roles in the xyloglucan-deficient walls (Park and Cosgrove, 2012b) which were mechanically weaker but simultaneously less extensible because they lacked the major target of α-expansin. Xyloglucan may also affect microfibril structure or aggregation (Atalla et al., 1993), with indirect consequences for the effectiveness of wall-loosening agents. This idea draws some support from NMR analyses (Dick-Perez et al., 2011) and confocal microscopy (Anderson et al., 2010) which indicate that the xyloglucan-deficient walls contain larger cellulose microfibrils or microfibril bundles compared with wildtype walls.

A revised view of xyloglucan’s role recently emerged from experiments in which substrate-specific endoglucanases were assessed for their ability to induce wall creep (Park and Cosgrove, 2012a). Although xyloglucan-specific endoglucanases removed much of the xyloglucan, they did not increase wall creep or compliance. Cellulose-specific endoglucanases were likewise inactive in biomechanical assays. Only enzymes able to hydrolyze both xyloglucan and cellulose were active in these assays, with large biomechanical effects associated with digestion of ~0.3% of the total xyloglucan. These results argue against the tether model (**Figure 1A**), as such tethers would be digested by xyloglucan-specific enzymes. **Figure 1B** shows one possible scheme consistent with the new results, where the biomechanically significant xyloglucan is restricted to inaccessible junctions between microfibrils.

These studies de-emphasize the role of xyloglucan as the exclusive moderator of cell wall mechanics and indicate that other matrix polysaccharides may fill in for some xyloglucan functions. This idea is further reinforced by studies showing pectin de-esterification is linked with enhanced growth in the *Arabidopsis* hypocotyl (Pelletier et al., 2010) and in the outgrowth of leaf and flower primordia in the shoot apical meristem (Peaucelle et al., 2008, 2011). These effects of pectin methyl esterase are hard to understand in terms of wall structure because pectin de-esterification, by itself, should lead to enhanced ionic cross linking of pectins, thereby rigidifying the wall (Zhao et al., 2008). Indirect effects of pectin de-esterification may be linked to these growth enhancements (Wolf and Greiner, 2012). Moreover, pectin may play a greater role in specialized walls found in pollen tubes and in some algae (Fayant et al., 2010; Rojas et al., 2011; Palin and Geitmann, 2012).

## BIOMECHANICS OF SECONDARY CELL WALLS

In most angiosperms the functions of water conduction and support are divided between vessels and interfascicular xylem cells, patterned through the influence of distinct transcription factors (Guo et al., 2009; Ohtani et al., 2011) and intercellular signaling networks (Fuchs et al., 2011). The requirement for tensile and compressive strength is satisfied by a high content of rather uniformly oriented cellulose, synthesized by a distinct set of cellulose synthases and normally lignified. In trees the result is wood (Mellerowicz and Sundberg, 2008). Xylem tissues broadly similar in structure and function, although often with less lignin or none (Carlquist and Schneider, 1998; Hepworth et al., 1998; Jung and Engels, 2002) are found in herbaceous plants, bamboos, and palms.

**FIGURE 2:**
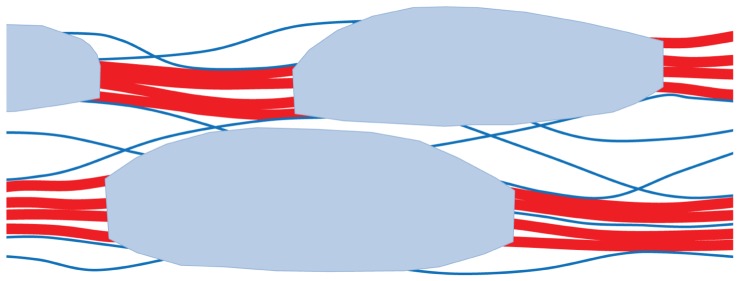
** Hypothetical architecture of polymer network in secondary cell walls of conifer wood**. Loosely aggregated bundles of cellulose microfibrils (red) are coated with a disordered xylan–lignin complex (shaded light blue). Partially oriented glucomannan chains (blue lines) adhere by hydrogen bonding to the cellulose aggregates and acetylated segments of these glucomannans bridge between the aggregates. For clarity the structure is shown much more open than is the case: the free space, filled by water *in vivo*, is only about 40% of the total volume. This is much less than the free space in hydrated primary cell walls. Based on Terashima et al. (2009) and Fernandes et al. (2011).

Like primary cell walls, secondary cell walls are composite materials based on 3-nm cellulose microfibrils, with lignin, xylans, and glucomannans replacing xyloglucans and pectins (Mellerowicz and Sundberg, 2008). Secondary cell walls are less hydrated than primary cell walls, containing only about 30% water at saturation. In coniferous wood at least, the cellulose microfibrils form loose bundles 10–20 nm across with direct lateral adhesion between adjacent microfibrils over part of their length (Fernandes et al., 2011). Most of the lignin and hemicelluloses are thought to lie outside these aggregates (Salmen, 2004; Fernandes et al., 2011), but hemicellulosic glucomannans are closely associated with cellulose (Salmen and Bergstrom, 2009; **Figure 2**). In contrast to the dispersed cellulose orientations in primary cell walls, the cellulose microfibrils are wound around wood cells in a helix whose pitch is defined by the microfibril angle, the angle that the microfibrils make with the cell axis (Barnett and Bonham, 2004). Tree saplings have high microfibril angles giving flexibility in response to wind and snow, while mature trees have low microfibril angles providing the stiffness to avoid buckling under the compressive loads imposed by their weight (Altaner and Jarvis, 2008). A tree’s mechanical history is recorded in the variation of microfibril angle across the annual rings of the trunk.

Because of the importance of wood as a construction material its mechanical properties have been studied much more intensively than those of growing plant cells (Salmen, 2004). Models and tentative mechanisms have been suggested for three kinds of deformation: (1) viscoelastic (reversible) deformation; (2) an irreversible time-dependent form of deformation termed here “viscoplastic” (Flores et al., 2011); (3) mechano-sorptive deformation. Only (2) and (3) are irreversible and therefore directly analogous to the growth of primary cell walls, but it is not clear to what extent (1)–(3) are mechanistically related. All three may therefore suggest insights or potential experimental approaches that might help to understand the physical processes occurring when primary cell walls elongate.

### VISCOELASTIC STRETCHING AND BENDING

Dry secondary cell walls with small microfibril angles show purely viscoelastic behavior, either in tensile or bending experiments at constant stress or by stress relaxation. The viscous component increases with the angle between the microfibrils and the stress (Bonfield et al., 1996; Gril et al., 2004) and has been modeled as due to viscous shear in the lignin–hemicellulose matrix between elastic microfibrils (Engelund and Svensson, 2011) or microfibril aggregates. The calculated free energy of activation was consistent with the breaking of four to six hydrogen bonds in each sliding event (Bonfield et al., 1996). Chemical delignification (Kohler and Spatz, 2002) or down regulation of lignin synthesis (Koehler and Telewski, 2006) generally reduced stiffness, demonstrating involvement of lignin. There was also less direct evidence for involvement of hemicelluloses (Assor et al., 2009; Bjurhager et al., 2010), but mechanisms remain unclear at the molecular level.

### VISCOPLASTIC DEFORMATION ABOVE THE YIELD THRESHOLD

When fully hydrated as in the living tree, wood with high microfibril angle shows a two-phase load-deformation curve. Above a threshold stress it becomes less stiff. This second phase of elongation is irreversible but this does not result from overt damage, because when the stress is reduced below the yield threshold the elongated sample remains as stiff as before (Kohler and Spatz, 2002; Keckes et al., 2003). Mechanically, then, elongation of wood above the yield threshold resembles turgor-driven extension growth as described by the Lockhart equation. The term “molecular Velcro” was coined to describe this form of deformation in wood because it is simulated by hook-and-loop fasteners that can be pulled apart, moved and refastened without permanent loss of strength (Keckes et al., 2003). Molecular Velcro tensile behavior has been demonstrated for wet wood samples in which the cellulose microfibrils are oriented at 20–45° to the cell axis. If the wood is dry or the microfibril angle is low, the yield threshold rises until the sample breaks before reaching it (Kamiyama et al., 2005). Because of this technical problem it is common to use isolated single wood cells, which can be stretched to greater elongation than bulk wood samples without breaking (Keckes et al., 2003). The helical arrangement of the microfibrils tends to untwist on stretching, so single wood cells need to be rotationally restrained to match their behavior in wood, where each cell is locked to the next and cannot twist (Fratzl et al., 2004).

The molecular Velcro phenomenon requires that cellulose microfibrils should slide past one another and should also rotate closer to the line of stress (Flores et al., 2011): these two deformations must be balanced if the cell is not to twist (Fratzl et al., 2004). The yield threshold then corresponds to the abrupt onset of sliding between aggregates of microfibrils. Several mechanisms have been suggested to trigger this sliding process. The original suggestion was that hemicelluloses anchored to two adjacent microfibrils were entangled and that a threshold shear stress was required to break the entanglement, allowing the microfibrils to slide (Keckes et al., 2003). There is doubt about whether the orientation of hemicelluloses is consistent with entanglement interactions (Salmen and Bergstrom, 2009), and a more recent explanation of this mechanism (Speck and Burgert, 2011) substitutes lateral association of two hemicellulose chains, stabilized by hydrogen bonding over a number of residues. A further alternative mechanism involves the peeling of a hemicellulose chain off the microfibril surface by the component of the stress perpendicular to the microfibril aggregates, allowing them to slide parallel to one another until contact is restored (Altaner and Jarvis, 2008). All these triggering mechanisms focus on hemicelluloses and assume topologies rather like the “tethered network” model for primary walls, but there is evidence for the involvement of lignin as well (Kohler and Spatz, 2002) as for viscoelastic extension below the yield stress.

### HYGROMECHANICAL DEFORMATION

Wood deforms progressively when maintained under a constant, often quite small, tensile or bending load through repeated cycles of drying and wetting (Bengtsson, 2001; Entwistle and Zadoroshnyj, 2008). The cumulative deformation is largely permanent unless the load is reversed. In that respect hygromechanical creep resembles viscoplastic deformation above the yield threshold, and it has been suggested to require breaking of hydrogen bonds between hemicelluloses and cellulose under hydrated conditions and their re-formation, in different locations, on drying (Entwistle and Zadoroshnyj, 2008).

All three of these forms of deformation of wood seem to be focused in the matrix region between microfibrils or microfibril aggregates, and increase simultaneously in magnitude with microfibril angle (Bengtsson, 2001; Fratzl et al., 2004; Gril et al., 2004), but it does not follow that they share exactly the same mechanism. There is an interesting parallel with the unexplained observation that stress relaxation parameters for the stretching of primary walls often correlate with growth (Nakamura et al., 2002).

## CONCLUSION

Although primary walls of growing cells possess inherent viscoelastic–viscoplastic properties arising from the polymeric nature of their structure, cell wall expansion is primarily a dynamic process requiring the action of expansins or other wall-loosening agents. Deformation of wood is considered to be physical. Nevertheless there are similarities. Both processes are sensitive to microfibril orientation and both involve interactions of hydrated non-cellulosic polysaccharides with cellulose surfaces. The topology of these interactions in response to the local distribution of stresses – details best conveyed in cartoons like **Figure 1** – are only now starting to be understood. In secondary cell walls the key structural elements are not individual microfibrils but bundles of microfibrils, and the possibility that this is also true in primary cell walls deserves to be explored. The research communities working on primary and secondary cell walls are rather separate, but both could benefit from converging and exchanging ideas.

## Conflict of Interest Statement

The authors declare that the research was conducted in the absence of any commercial or financial relationships that could be construed as a potential conflict of interest.

## References

[B1] Abasolo W. Eder M. Yamauchi K. Obel N. Reinecke A. Neumetzler L. Dunlop J. W. Mouille G. Pauly M. Hofte H. Burgert I. (2009). Pectin may hinder the unfolding of xyloglucan chains during cell deformation: implications of the mechanical performance of Arabidopsis hypocotyls with pectin alterations. *Mol. Plant* 2 990–999 19825674 10.1093/mp/ssp065

[B2] Altaner C. M. Jarvis M. C. (2008). Modelling polymer interactions of the “molecular Velcro” type in wood under mechanical stress. *J. Theor. Biol.* 253 434–445 18485371 10.1016/j.jtbi.2008.03.010

[B3] Anderson C. T. Carroll A. Akhmetova L. Somerville C. (2010). Real-time imaging of cellulose reorientation during cell wall expansion in *Arabidopsis* roots. *Plant Physiol.* 152 787–796 19965966 10.1104/pp.109.150128 PMC2815888

[B4] Assor C. Placet V. Chabbert B. Habrant A. Lapierre C. Pollet B. Perre P. (2009). Concomitant changes in viscoelastic properties and amorphous polymers during the hydrothermal treatment of hardwood and softwood. *J. Agric. Food Chem.* 57 6830–6837 19618934 10.1021/jf901373s

[B5] Atalla R. H. Hackney J. M. Uhlin I. Thompson N. S. (1993). Hemicelluloses as structure regulators in the aggregation of native cellulose. *Int. J. Biol. Macromol.* 15 109–112 8485102 10.1016/0141-8130(93)90007-9

[B6] Barnett J. R. Bonham V. A. (2004). Cellulose microfibril angle in the cell wall of wood fibres. *Biol. Rev.* 79 461–472 15191232 10.1017/s1464793103006377

[B7] Baskin T. I. (2005). Anisotropic expansion of the plant cell wall. *Annu. Rev. Cell Dev. Biol.* 21 203–222 16212493 10.1146/annurev.cellbio.20.082503.103053

[B8] Bengtsson C. (2001). Mechano-sorptive bending creep of timber – influence of material parameters. *Holz Roh Werkst.* 59 229–236

[B9] Bergenstrahle M. Thormann E. Nordgren N. Berglund L. A. (2009). Force pulling of single cellulose chains at the crystalline cellulose-liquid interface: a molecular dynamics study. *Langmuir* 25 4635–4642 19231815 10.1021/la803915c

[B10] Bjurhager I. Olsson A. M. Zhang B. Gerber L. Kumar M. Berglund L. A. Burgert I. Sundberg B. Salmen L. (2010). Ultrastructure and mechanical properties of *Populus* wood with reduced lignin content caused by transgenic down-regulation of cinnamate 4-hydroxylase. *Biomacromolecules* 11 2359–2365 20831275 10.1021/bm100487e

[B11] Bonfield P. W. Mundy J. Robson D. J. Dinwoodie J. M. (1996). The modelling of time-dependant deformation in wood using chemical kinetics. *Wood Sci. Technol.* 30 105–115

[B12] Bootten T. J. Harris P. J. Melton L. D. Newman R. H. (2004). Solid-state 13C-NMR spectroscopy shows that the xyloglucans in the primary cell walls of mung bean (*Vigna radiata* L.) occur in different domains: a new model for xyloglucan–cellulose interactions in the cell wall. *J. Exp. Bot.* 55 571–583 14966211 10.1093/jxb/erh065

[B13] Carlquist S. Schneider E. L. (1998). Origin and nature of vessels in monocotyledons. 5. Araceae subfamily Colocasioideae. *Bot. J. Linn. Soc.* 128 71–86

[B14] Carpita N. C. Defernez M. Findlay K. Wells B. Shoue D. A. Catchpole G. Wilson R. H. McCann M. C. (2001). Cell wall architecture of the elongating maize coleoptile. *Plant Physiol.* 127 551–565 11598229 PMC125090

[B15] Carpita N. C. Gibeaut D. M. (1993). Structural models of primary cell walls in flowering plants: consistency of molecular structure with the physical properties of the walls during growth. *Plant J.* 3 1–30 8401598 10.1111/j.1365-313x.1993.tb00007.x

[B16] Cavalier D. M. Lerouxel O. Neumetzler L. Yamauchi K. Reinecke A. Freshour G. Zabotina O. A. Hahn M. G. Burgert I. Pauly M. Raikhel N. V. Keegstra K. (2008). Disrupting two *Arabidopsis thaliana* xylosyltransferase genes results in plants deficient in xyloglucan, a major primary cell wall component. *Plant Cell* 20 1519–1537 18544630 10.1105/tpc.108.059873 PMC2483363

[B17] Cosgrove D. J. (1993). Wall extensibility: its nature, measurement, and relationship to plant cell growth. *New Phytol.* 124 1–23 11537718 10.1111/j.1469-8137.1993.tb03795.x

[B18] Cosgrove D. J. (2005). Growth of the plant cell wall. *Nat. Rev. Mol. Cell Biol.* 6 850–861 16261190 10.1038/nrm1746

[B19] Dick-Perez M. Zhang Y. Hayes J. Salazar A. Zabotina O. A. Hong M. (2011). Structure and interactions of plant cell-wall polysaccharides by two- and three-dimensional magic-angle-spinning solid-state NMR. *Biochemistry* 50 989–1000 21204530 10.1021/bi101795q

[B20] Dyson R. J. Band L. R. Jensen O. E. (2012). A model of crosslink kinetics in the expanding plant cell wall: yield stress and enzyme action. *J. Theor. Biol.* 307C 125–136 22584249 10.1016/j.jtbi.2012.04.035 PMC3414840

[B21] Dyson R. J. Jensen O. E. (2010). A fibre-reinforced fluid model of anisotropic plant cell growth. *J. Fluid Mech.* 655 472–503

[B22] Eklof J. M. Brumer H. (2010). The XTH gene family: an update on enzyme structure, function, and phylogeny in xyloglucan remodeling. *Plant Physiol.* 153 456–466 20421457 10.1104/pp.110.156844 PMC2879796

[B23] Engelund E. T. Svensson S. (2011). Modelling time-dependent mechanical behaviour of softwood using deformation kinetics. *Holzforschung* 65 231–237

[B24] Entwistle K. M. Zadoroshnyj A. (2008). The recovery of mechano-sorptive creep strains. *J. Mater. Sci.* 43 967–973

[B25] Fayant P. Girlanda O. Chebli Y. Aubin C.-E. Villemure I. Geitmann A. (2010). Finite element model of polar growth in pollen tubes. *Plant Cell* 22 2579–2593 20699395 10.1105/tpc.110.075754 PMC2947179

[B26] Fernandes A. N. Thomas L. H. Altaner C. M. Callow P. Forsyth V. T. Apperley D. C. Kennedy C. J. Jarvis M. C. (2011). Nanostructure of cellulose microfibrils in spruce wood. *Proc. Natl. Acad. Sci. U.S.A.* 108 E1195–E1203 22065760 10.1073/pnas.1108942108 PMC3223458

[B27] Flores E. I. S. Neto E. A. D. Pearce C. (2011). A large strain computational multi-scale model for the dissipative behaviour of wood cell-wall. *Comput. Mater. Sci.* 50 1202–1211

[B28] Fratzl P. Burgert I. Keckes J. (2004). Mechanical model for the deformation of the wood cell wall. *Z. Metallkd.* 95 579–584

[B29] Fuchs M. Van Bel A. J. E. Ehlers K. (2011). Do symplasmic networks in cambial zones correspond with secondary growth patterns? *Protoplasma* 248 141–151 20853011 10.1007/s00709-010-0208-7

[B30] Geitmann A. (2010). Mechanical modeling and structural analysis of the primary plant cell wall. *Curr. Opin. Plant Biol.* 13 693–699 20971032 10.1016/j.pbi.2010.09.017

[B31] Gibeaut D. M. Pauly M. Bacic A. Fincher G. B. (2005). Changes in cell wall polysaccharides in developing barley (*Hordeum vulgare*) coleoptiles. *Planta* 221 729–738 15824908 10.1007/s00425-005-1481-0

[B32] Gril J. Hunt D. Thibaut B. (2004). Using wood creep data to discuss the contribution of cell-wall reinforcing material. *C. R. Biol.* 327 881–888 15587079 10.1016/j.crvi.2004.08.002

[B33] Guo Y. Qin G. J. Gu H. Y. Qu L. J. (2009). Dof5.6/HCA2, a Dof transcription factor gene, regulates interfascicular cambium formation and vascular tissue development in *Arabidopsis*. *Plant Cell* 21 3518–3534 19915089 10.1105/tpc.108.064139 PMC2798324

[B34] Hamant O. Traas J. (2010). The mechanics behind plant development. *New Phytol.* 185 369–385 20002316 10.1111/j.1469-8137.2009.03100.x

[B35] Hayashi T. Kaida R. (2011). Functions of xyloglucan in plant cells. *Mol. Plant* 4 17–24 20943810 10.1093/mp/ssq063

[B36] Hepworth D. G. Vincent J. F. V. Schuch W. (1998). Using viscoelastic properties of the woody tissue from tobacco plants (*Nicotiana tabacum*) to comment on the molecular structure of cell walls. *Ann. Bot.* 81 729–734

[B37] Jung H. G. Engels F. M. (2002). Alfalfa stem tissues: cell wall deposition, composition, and degradability. *Crop Sci.* 42 524–534

[B38] Kamiyama T. Suzuki H. Sugiyama J. (2005). Studies of the structural change during deformation in *Cryptomeria japonica* by time-resolved synchrotron small-angle X-ray scattering. *J. Struct. Biol.* 151 1–11 15963733 10.1016/j.jsb.2005.04.007

[B39] Keckes J. Burgert I. Fruhmann K. Muller M. Kolln K. Hamilton M. Burghammer M. Roth S. V. Stanzl-Tschegg S. Fratzl P. (2003). Cell-wall recovery after irreversible deformation of wood. *Nat. Mater.* 2 810–814 14625541 10.1038/nmat1019

[B40] Keegstra K. Talmadge K. W. Bauer W. D. Albersheim P. (1973). The structure of plant cell walls. III. A model of the walls of suspension-cultured sycamore cells based on the interconnections of the macromolecular components. *Plant Physiol.* 51 188–196 10.1104/pp.51.1.188 PMC367377 16658282

[B41] Kerstens S. Decraemer W. F. Verbelen J.-P. (2001). Cell walls at the plant surface behave mechanically like fiber-reinforced composite materials. *Plant Physiol.* 127 381–385 11598213 PMC1540143

[B42] Kha H. Tuble S. C. Kalyanasundaram S. Williamson R. E. (2010). WallGen, software to construct layered cellulose–hemicellulose networks and predict their small deformation mechanics. *Plant Physiol.* 152 774–786 20007450 10.1104/pp.109.146936 PMC2815898

[B43] Koch G. W. Sillett S. C. Jennings G. M. Davis S. D. (2004). The limits to tree height. *Nature* 428 851–854 15103376 10.1038/nature02417

[B44] Koehler L. Telewski F. W. (2006). Biomechanics and transgenic wood. *Am. J. Bot.* 93 1433–1438 21642090 10.3732/ajb.93.10.1433

[B45] Kohler L. Spatz H. C. (2002). Micromechanics of plant tissues beyond the linear-elastic range. *Planta* 215 33–40 12012239 10.1007/s00425-001-0718-9

[B46] Lockhart J. A. (1965). An analysis of irreversible plant cell elongation. *J. Theor. Biol.* 8 264–275 5876240 10.1016/0022-5193(65)90077-9

[B47] Maris A. Suslov D. Fry S. C. Verbelen J. P. Vissenberg K. (2009). Enzymic characterization of two recombinant xyloglucan endotransglucosylase/hydrolase (XTH) proteins of *Arabidopsis* and their effect on root growth and cell wall extension. *J. Exp. Bot.* 60 3959–3972 19635745 10.1093/jxb/erp229

[B48] Mellerowicz E. J. Sundberg B. (2008). Wood cell walls: biosynthesis, developmental dynamics and their implications for wood properties. *Curr. Opin. Plant Biol.* 11 293–300 18434240 10.1016/j.pbi.2008.03.003

[B49] Miedes E. Zarra I. Hoson T. Herbers K. Sonnewald U. Lorences E. P. (2011). Xyloglucan endotransglucosylase and cell wall extensibility. *J. Plant Physiol.* 168 196–203 20828871 10.1016/j.jplph.2010.06.029

[B50] Nakamura Y. Wakabayashi K. Kamisaka S. Hoson T. (2002). Effects of temperature on the cell wall and osmotic properties in dark-grown rice and azuki bean seedlings. *J. Plant Res.* 115 455–461 12579449 10.1007/s10265-002-0058-2

[B51] Ohtani M. Nishikubo N. Xu B. Yamaguchi M. Mitsuda N. Goue N. Shi F. Ohme-Takagi M. Demura T. (2011). A NAC domain protein family contributing to the regulation of wood formation in poplar. *Plant J.* 67 499–512 21649762 10.1111/j.1365-313X.2011.04614.x

[B52] Palin R. Geitmann A. (2012). The role of pectin in plant morphogenesis. *Biosystems.* 10.1016/j.biosystems.2012.04.006 [Epub ahead of print]. 22554809

[B53] Park Y. B. Cosgrove D. J. (2012a). A revised architecture of primary cell walls based on biomechanical changes induced by substrate-specific endoglucanases. *Plant Physiol.* 158 1933–1943 22362871 10.1104/pp.111.192880 PMC3320196

[B54] Park Y. B. Cosgrove D. J. (2012b). Changes in cell wall biomechanical properties in the xyloglucan-deficient *xxt1/xxt2* mutant of *Arabidopsis*. *Plant Physiol.* 158 465–475 22108526 10.1104/pp.111.189779 PMC3252101

[B55] Peaucelle A. Braybrook S. A. Le Guillou L. Bron E. Kuhlemeier C. Hofte H. (2011). Pectin-induced changes in cell wall mechanics underlie organ initiation in *Arabidopsis*. *Curr. Biol.* 21 1720–1726 21982593 10.1016/j.cub.2011.08.057

[B56] Peaucelle A. Louvet R. Johansen J. N. Hofte H. Laufs P. Pelloux J. Mouille G. (2008). *Arabidopsis* phyllotaxis is controlled by the methyl-esterification status of cell-wall pectins. *Curr. Biol.* 18 1943–1948 19097903 10.1016/j.cub.2008.10.065

[B57] Pelletier S. Van Orden J. Wolf S. Vissenberg K. Delacourt J. Ndong Y. A. Pelloux J. Bischoff V. Urbain A. Mouille G. Lemonnier G. Renou J. P. Hofte H. (2010). A role for pectin de-methylesterification in a developmentally regulated growth acceleration in dark-grown *Arabidopsis* hypocotyls. *New Phytol.* 188 726–739 20819179 10.1111/j.1469-8137.2010.03409.x

[B58] Preston R. D. (1974). *The Physical Biology of Plant Cell Walls*. London: Chapman & Hall

[B59] Ray P. M. Ruesink A. W. (1962). Kinetic experiments on the nature of the growth mechanism in oat coleoptile cells. *Dev. Biol.* 4 377–397

[B60] Rojas E. R. Hotton S. Dumais J. (2011). Chemically mediated mechanical expansion of the pollen tube cell wall. *Biophys. J*. 101 1844–1853 22004737 10.1016/j.bpj.2011.08.016 PMC3192986

[B61] Saladie M. Rose J. K. Cosgrove D. J. Catala C. (2006). Characterization of a new xyloglucan endotransglucosylase/hydrolase (XTH) from ripening tomato fruit and implications for the diverse modes of enzymic action. *Plant J.* 47 282–295 16774648 10.1111/j.1365-313X.2006.02784.x

[B62] Salmen L. (2004). Micromechanical understanding of the cell-wall structure. *C. R. Biol.* 327 873–880 15587078 10.1016/j.crvi.2004.03.010

[B63] Salmen L. Bergstrom E. (2009). Cellulose structural arrangement in relation to spectral changes in tensile loading FTIR. *Cellulose* 16 975–982

[B64] Sampedro J. Cosgrove D. J. (2005). The expansin superfamily. *Genome Biol.* 6 242 10.1186/gb-2005-6-12-242 PMC1414085 16356276

[B65] Scheller H. V. Ulvskov P. (2010). Hemicelluloses. *Annu. Rev. Plant Biol.* 61 263–289 20192742 10.1146/annurev-arplant-042809-112315

[B66] Speck T. Burgert I. (2011). Plant stems: functional design and mechanics. *Annu. Rev. Mater. Res.* 41 169–193

[B67] Terashima N. Kitano K. Kojima M. Yoshida M. Yamamoto H. Westermark U. (2009). Nanostructural assembly of cellulose, hemicellulose, and lignin in the middle layer of secondary wall of ginkgo tracheid. *J. Wood Sci.* 55 409–416

[B68] Thimm J. C. Burritt D. J. Sims I. M. Newman R. H. Ducker W. A. Melton L. D. (2002). Celery (*Apium graveolens*) parenchyma cell walls: cell walls with minimal xyloglucan. *Physiol. Plant.* 116 164–171 12354192 10.1034/j.1399-3054.2002.1160205.x

[B69] Thompson D. S. (2005). How do cell walls regulate plant growth? *J. Exp. Bot.* 56 2275–2285 16061505 10.1093/jxb/eri247

[B70] Veytsman B. A. Cosgrove D. J. (1998). A model of cell wall expansion based on thermodynamics of polymer networks. *Biophys. J.* 75 2240–2250 9788919 10.1016/S0006-3495(98)77668-4 PMC1299898

[B71] Wolf S. Greiner S. (2012). Growth control by cell wall pectins. *Protoplasma* 249(Suppl. 2) 169–175 10.1007/s00709-011-0371-5 22215232

[B72] Yennawar N. H. Li L. C. Dudzinski D. M. Tabuchi A. Cosgrove D. J. (2006). Crystal structure and activities of EXPB1 (Zea m 1), a β-expansin and group-1 pollen allergen from maize. *Proc. Natl. Acad. Sci. U.S.A.* 103 14664–14671 16984999 10.1073/pnas.0605979103 PMC1595409

[B73] Zabotina O. A. Avci U. Cavalier D. Pattathil S. Chou Y. H. Eberhard S. Danhof L. Keegstra K. Hahn M. G. (2012). Mutations in multiple *XXT* genes of *Arabidopsis* reveal the complexity of xyloglucan biosynthesis. *Plant Physiol.* 159 1367–1348 22696020 10.1104/pp.112.198119 PMC3425184

[B74] Zhao Q. Yuan S. Wang X. Zhang Y. Zhu H. Lu C. (2008). Restoration of mature etiolated cucumber hypocotyl cell wall susceptibility to expansin by pretreatment with fungal pectinases and EGTA in vitro. *Plant Physiol.* 147 1874–1885 18562768 10.1104/pp.108.116962 PMC2492596

[B75] Zykwinska A. Gaillard C. Buleon A. Pontoire B. Garnier C. Thibault J. F. Ralet M. C. (2007a). Assessment of in vitro binding of isolated pectic domains to cellulose by adsorption isotherms, electron microscopy, and X-ray diffraction methods. *Biomacromolecules* 8 223–232 17206811 10.1021/bm060292h

[B76] Zykwinska A. Thibault J. F. Ralet M. C. (2007b). Organization of pectic arabinan and galactan side chains in association with cellulose microfibrils in primary cell walls and related models envisaged. *J. Exp. Bot.* 58 1795–1802 17383990 10.1093/jxb/erm037

[B77] Zykwinska A. Thibault J. F. Ralet M. C. (2008). Competitive binding of pectin and xyloglucan with primary cell wall cellulose. *Carbohydr. Polym.* 74 957–961

